# Pushing the accuracy limit of shape complementarity for protein-protein docking

**DOI:** 10.1186/s12859-019-3270-y

**Published:** 2019-12-24

**Authors:** Yumeng Yan, Sheng-You Huang

**Affiliations:** School of Physics, Huazhong University of Science and Technology, Luoyu Road 1037, Wuhan, Hubei, 430074 P.R. China

**Keywords:** Molecular docking, Shape complementarity, Protein-protein Interactions, Scoring function, Fast-Fourier transform

## Abstract

**Background:**

Protein-protein docking is a valuable computational approach for investigating protein-protein interactions. Shape complementarity is the most basic component of a scoring function and plays an important role in protein-protein docking. Despite significant progresses, shape representation remains an open question in the development of protein-protein docking algorithms, especially for grid-based docking approaches.

**Results:**

We have proposed a new pairwise shape-based scoring function (LSC) for protein-protein docking which adopts an exponential form to take into account long-range interactions between protein atoms. The LSC scoring function was incorporated into our FFT-based docking program and evaluated for both bound and unbound docking on the protein docking benchmark 4.0. It was shown that our LSC achieved a significantly better performance than four other similar docking methods, ZDOCK 2.1, MolFit/G, GRAMM, and FTDock/G, in both success rate and number of hits. When considering the top 10 predictions, LSC obtained a success rate of 51.71% and 6.82% for bound and unbound docking, respectively, compared to 42.61% and 4.55% for the second-best program ZDOCK 2.1. LSC also yielded an average of 8.38 and 3.94 hits per complex in the top 1000 predictions for bound and unbound docking, respectively, followed by 6.38 and 2.96 hits for the second-best ZDOCK 2.1.

**Conclusions:**

The present LSC method will not only provide an initial-stage docking approach for post-docking processes but also have a general implementation for accurate representation of other energy terms on grids in protein-protein docking. The software has been implemented in our HDOCK web server at http://hdock.phys.hust.edu.cn/.

## Background

As one of the most fundamental organic macromolecules in living systems, proteins are involved in many biological processes like signal transduction, immune recognition, and intracellular trafficking. [1–12]. Therefore, the atomic structures of protein-protein complexes are valuable to investigate the interaction mechanism and thus develop potential drugs. [13–16]. With the rapid development of structural proteomics project in the past decades, the 3D structures of many proteins have been solved and deposited in the Protein Data Bank(PDB) [17]. Nevertheless, due to the technical difficulties and high cost of experimental approaches, the number of complex structures is still very limited compared to the number of individual proteins in the PDB [18]. Therefore, computational methods like protein-protein docking, which predicts the complex structures from individual proteins, have become an important complement of experimental approaches in determining the structures of protein-protein complexes [1, 13, 19–23].

For years, a number of protein-protein docking algorithms with different speed and accuracy have been developed [13, 22, 24–29]. Current protein docking methods can be grouped into three broad categories according to their different sampling strategies: direct search, fast-Fourier transform (FFT)-based search, and post-docking methods [13]. In direct search methods, putative binding modes are directly sampled in real space, i.e. Cartesian space. The sampling process can be local or global [30–39] depending on the availability of information about the binding site. FFT-based docking is a grid-based algorithm and was first proposed by Katchalski-Katzir et al. [40], in which the search for binding modes is accelerated by an FFT algorithm in three-dimensional translational space and thus the computational time was reduced from *O*(*N*^6^) to *O*(*N*^3^ log(*N*^3^)) in global sampling [40–51]. Due to its fast global search, many FFT-based protein-protein docking algorithms have been developed in the past decade and achieved considerable successes in the community-wide CAPRI (Critical Assessment of Prediction of Interactions) experiments (http://capri.ebi.ac.uk/) [52–57]. Post-docking algorithms are designed to improve the ranking/quality of correct binding modes by refining the putative modes obtained from other sampling strategies [58–64]. As the biological information and protein flexibility can be conveniently incorporated in a small number of docking solutions, post-docking refinement/filtering has been become a common procedure during protein-protein docking processes and received significant successes in the field [26, 52–56, 65].

Scoring function is essential for all docking algorithms to evaluate and rank the sampled conformations. Shape complementarity is the most basic component of scoring function [20, 47, 66, 67], and plays a vital role in searching putative binding poses [13, 68, 69] and ranking the sampled poses [13, 20, 22]. As for FFT-based docking algorithms, shape complementarity is particularly crucial because in addition to serving as a basic scoring element, it also influences the grid discretization of other energy terms [13]. As such, various approaches have been developed to characterize the shape complementarity in existing docking programs. For the direct search in real space, docking algorithms normally use graphics-based algorithms like distance geometry and Geometry Hashing to search the curvature-dependent shape complementarity between molecular surfaces [70–75]. For FFT-based algorithms, most docking programs simply try to find the optimal matches between the surface layers minus the clash penalty between the protein cores by mapping proteins onto grids, which we call grid-based shape complementarity function (GSC) [40]. To include information regarding surface curvature, the Weng group has presented a state-of-art pairwise shape complementarity (PSC) scoring function to reward the close atomic contacts between the receptor and the ligand [47], which significantly improved the docking performance.

**Fig. 1:**
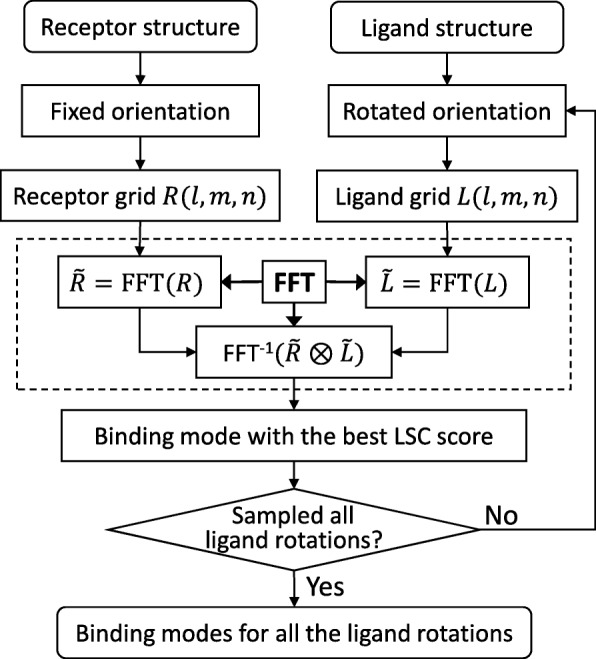
A flowchart of our FFT-based docking algorithm

Despite the significant progresses in current shape complementarity functions, all of them just simply consider the effects of neighboring atoms for a grid point, as we can see from the Fig 1 in the Weng’s pervious study [47]. However, some shape-based interactions like van der Waals interactions involve not only the nearest-neighboring atoms, but also many more other non nearest-neighboring interactions. Therefore, we have here presented a new pairwise scoring function for our FFT-based docking algorithm, which we call LSC, to consider the long range effect of protein atoms by an exponential form. Our docking algorithm HDOCK with LSC has been extensively tested on the protein-protein docking benchmark 4.0 [76]. The docking results have been compared with four other shape-based scoring methods that use GSC or PSC scoring function and simply consider the nearest-neighboring atoms. The comparison has shown the significant improvement of our LSC for both the success rate and the number of hits in predicting binding modes for both bound and unbound docking. Our FFT-based algorithm with LSC may act as the initial stage of hybrid docking strategy for post-docking algorithms. Moreover, as shape complementarity is important for characterizing other energy terms like desolvation, electrostatics and hydrogen bonding, our LSC method is also expected to be useful in developing accurate docking/scoring algorithms.

## Materials and methods

### Sampling strategy

We have used a global search approach to sample putative binding modes in our docking algorithm, as shown in Fig. 1, which is similar to that in other FFT-based docking methods. Specifically, the receptor protein is fixed and the ligand protein is rotated in the rotational space by an interval of Euler angles (*Δθ*,*Δϕ*,*Δψ*). Then the receptor and the ligand are discretized into grids and for each rotation, 3D FFT is used to accelerate the calculation of shape complementarity scores between the receptor and ligand grids During the rotational sampling and translational search of the docking process, an angle interval of 15^∘^ is adopted and a grid spacing of 1.2 Å is used respectively. After sampling the rotation by uniformly distributed Euler angles, 4392 orientations in the rotational space are obtained. For each rotation of the ligand, the translation with the best shape complementarity score is retained, which yields a total of 4392 predicted models for a global docking run.

### FFT-based docking with lSC

To perform the FFT algorithm, both the receptor and ligand proteins are first discretized onto a three dimensional grid of *N*×*N*×*N* points [40, 47]. If the grid points are within the VDW radius of any protein atoms, they are defined as inside the protein; otherwise, they are considered as outside the protein. The VDW radii of protein atoms with different type are derived from literature [77]. Then, the grid points inside the protein are divided into three parts: core region, near-surface layer and surface layer. If any of neighboring grid point is outside the protein, the grid point is defined as in the surface layer. Similarly, if any of neighbors belongs to the surface layer, the grid point is considered as the near-surface-layer grid point. Finally, all the remaining inside-protein grid points are defined as the core region. According to the description above, the core region and near-surface layer are usually occupied by protein atoms, and the surface layer are the spacer layer which separates the inside of the protein from the outside. Then, each receptor (R) and ligand (L) grid point is assigned a complex value as:

1 $$\begin{array}{@{}rcl@{}} R(l,m,n)= \left \{ \begin{array}{llll} -\sum\limits_{i,j,k}\exp[-(r-1)^{2}]{+}J & \mathrm{for\ the\ surface\ layer} \\ -1{+}2J\times\sum\limits_{i,j,k}\exp(-r^{2}) & \mathrm{ for\ the\ near\ surface\ layer} \\ -1{+}10J & \text{for\ the\ core} \\ 0 & \text{outside\ the\ protein} \\ \end{array} \right. \end{array} $$

and

2 $$\begin{array}{@{}rcl@{}} L(l,m,n)= \left \{ \begin{array}{llll} 1{-}J & \mathrm{ for\ the\ surface\ layer} \\ 1{-}2J\times\sum\limits_{i,j,k}\exp(-r^{2}) & \mathrm{ for\ the\ near\ surface\ layer} \\ 1{-}10J & \text{for\ the\ core} \\ 0 & \text{outside\ the\ protein} \\ \end{array} \right. \end{array} $$

where *J*^2^=−1, *l*, *m*, and *n* are the 3D indices of the grid (*l*,*m*,*n*=1,⋯,*N*), and *r* is the distance between the grid points of (*i*,*j*,*k*) and (*l*,*m*,*n*). Here, for the near-surface layer, *i*∈[*l*−1,*l*+1],*j*∈[*m*−1,*m*+1], and *k*∈[*n*−1,*n*+1], and for the surface layer, *i*∈[*l*−3,*l*+3],*j*∈[*m*−3,*m*+3], and *k*∈[*n*−3,*n*+3]. In addition, the grid point (*i*,*j*,*k*) should belong to the near-surface layer or the protein core. Here, up to 7 neighbouring layers are considered for the surface and up to 3 neighbouring layers are considered for the near surface to take into account the effects of the long range interactions, while the GSC and PSC only consider the nearest layer.

On the basis of grid discretization of the proteins, the shape complementarity score of a receptor-ligand complex can be calculated using the following formula [40, 47]

3 $$\begin{array}{@{}rcl@{}}  {\begin{array}{l} E(o,p,q) =\\ \text{Re} \left[ \sum\limits^{N}_{l=1}\sum\limits^{N}_{m=1}\sum\limits^{N}_{n=1} {R(l,m,n)\times L(l+o,m+p,n+q)}\right] \end{array}} \end{array} $$

where Re[*x*] stands for the real part of a complex number *x*, *N*×*N*×*N* is the size of the receptor and ligand grid box, and *o*, *p*, and *q* are the numbers of shifted grid points in three translational dimensions of the ligand (L) relative to the receptor (R). Namely, *o*, *p*, and *q* are the moved translational distances, in the *x*,*y*,*z* dimensions of the lattice, respectively. If the index *l*+*o*,*m*+*p*, or *n*+*q* is larger than *N*, it will take the number after subtracting *N* from itself. The calculation of Eq. (3) can be accelerated by 3D FFT. For a translation of (*o*,*p*,*q*), higher correlation score means better shape complementarity between the receptor and ligand grids.

Repeating the FFT calculation of Eq. (3) for all the ligand rotations, our docking program can perform the exhaustive global sampling in the six (three translational + three rotational) degrees of freedom of the search space. A docking calculation can be completed in 10 mins on average on a 2.6 GHZ Intel CPU core which shows the computational efficiency of our FFT-based docking program.

### Test set

The protein-protein docking benchmark 4.0 constructed by the Weng’s group [76] was used to evaluate our FFT-based docking program with LSC. There are a total of 176 diverse targets in the benchmark, which contains 52 enzyme-inhibitor cases (EI), 25 antibody-antigen cases (AA), and 99 cases of other types (OT). Each target consists of the co-crystalized bound structures and their corresponding unbound structures for the receptor and the ligand. The unbound structures are superimposed onto their respective bound structures for the convenience of evaluation. The benchmark has been extensively used to evaluate the performance of docking algorithms and scoring functions [22].

### Evaluation criteria

Similar to previous studies [22], the ligand root mean square deviation (*L*_rmsd_) was adopted to evaluate the quality of predicted models, and it was calculated based on the *Cα* atoms of the ligand between the predicted mode and the native structure after the receptor proteins were superimposed according to their backbone atoms. A predicted binding pose with an *L*_rmsd_ less than 10 Å was considered as a successful prediction or a ‘hit’. The success rate was used to assess the performance of a scoring function in binding mode predictions, which was defined as the percentage of the test cases in benchmark with at least one hit when a certain number of top predictions were considered.

## Results

### Bound docking

We first performed bound docking with our LSC scoring function on the protein docking benchmark 4.0. As there is no conformational change in the bound structures, so bound docking can serves as a first-step to evaluate the performance of a docking/scoring algorithm. A reasonable scoring function should preform well in bound docking.

**Fig. 2:**
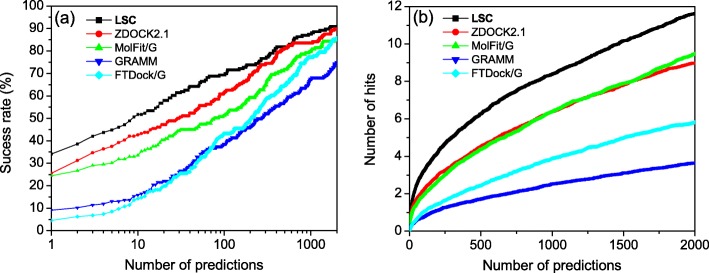
The success rate (**a**) and the average number of hits per target (**b**) as a function of the number of top predictions for our LSC-implemented docking program, ZDOCK 2.1, MolFit/G, GRAMM, and FTDock/G for bound docking on the protein-protein docking benchmark 4.0. The results of MolFit/G, GRAMM, and FTDock/G were taken from our previous study [22]

The results of bound docking with the success rate and the average number of hits per case as a function of the number of top predictions by our LSC method are shown in Fig. 2. Tables 1 and 2 list the values of success rate and average number of hits for several certain numbers of top predictions. For comparison, the corresponding results of four other shape-based docking programs, ZDOCK 2.1 [47], MolFit/G [45], GRAMM [42], and FTDock/G [41], are also shown in Fig. 2 and Tables 1-2. Here, a grid-based shape complementarity (GSC) scoring function is used in GRAMM and FTDock/G, while a pairwise shape complementarity (PSC) function is adopted by ZDOCK 2.1 in docking. From Fig. 2a we can see that our LSC method performed better than the other four docking/scoring programs in binding mode predictions. Our LSC method obtained a success rate of 34.09, 51.71, 69.32, and 87.50% for top 1, 10, 100 and 1000 predictions respectively, while ZDOCK 2.1 achieved a success rate of 25.57, 42.61, 61.36, and 83.52%, followed by 24.43, 33.52, 51.14, and 79.55% for MolFit/G, 9.09, 15.91, 38.64, 67.61% for GRAMM, and 4.55, 14.21, 43.18, and 77.27 for FTDock/G correspondingly (Table 1). As for the average number of hits, our LSC also achieved a better performance compared to the other four methods. For top 100, 500, 1000 and 2000 predictions, our LSC obtained an average of 3.21, 6.26, 8.38, and 11.64 hits per complex, compared to 2.18, 4.51, 6.38, and 8.97 hits for ZDOCK 2.1, 1.96, 4.36, 6.37, and 9.47 hits for MolFit/G, 0.76, 1.73, 2.54, and 3.66 hits for GRAMM, and 1.05, 2.43, 3.87, and 5.82 hits for FTDock/G (Fig. 2b and Table 2). The considerably better results of our LSC than similar methods for bound docking suggests that it is important and necessary to consider the long range interactions when using the shape complementarity scoring function in protein-protein docking.

**Table 1:** The success rates (%) predicted by our docking program with LSC and four other docking approaches with shape-based scoring functions on the protein docking benchmark 4.0 of 176 test cases when the top 1, 10, 100, and 100 predictions were considered

Method	Bound docking					Unbound docking			
	1	10	100	1000		1	10	100	1000
LSC	34.09	51.71	69.32	87.50		2.84	6.82	26.14	55.11
ZDOCK 2.1	25.57	42.61	61.36	83.52		1.71	4.55	23.86	48.86
MolFit/G	24.43	33.52	51.14	79.55		1.14	2.84	18.75	46.02
GRAMM	9.09	15.91	38.64	67.61		0.00	2.84	10.80	30.68
FTDock/G	4.55	14.21	43.18	77.27		0.57	1.71	11.36	42.61

**Table 2:** The average number of hits per complex obtained by our docking algorithm with LSC and four other docking approaches with shape-based scoring functions on the protein docking benchmark 4.0 of 176 test cases when the top 100, 500, 1000, and 2000 predictions were considered

Method	Bound docking					Unbound docking			
	100	500	1000	2000		100	500	1000	2000
LSC	3.21	6.26	8.38	11.64		0.83	2.54	3.94	6.51
ZDOCK 2.1	2.18	4.51	6.38	8.97		0.61	1.89	2.96	4.84
MolFit/G	1.96	4.36	6.37	9.47		0.36	1.47	2.62	4.55
GRAMM	0.76	1.73	2.54	3.66		0.18	0.51	0.86	1.63
FTDock/G	1.05	2.43	3.87	5.82		0.18	0.65	1.13	2.08

### Unbound docking

We have further evaluated our docking algorithm with LSC on the unbound structures of the 176 cases in the benchmark. Although bound docking is a more suitable way to test the scoring function, unbound docking is more realistic as only unbound structures are available in real applications.

**Fig. 3:**
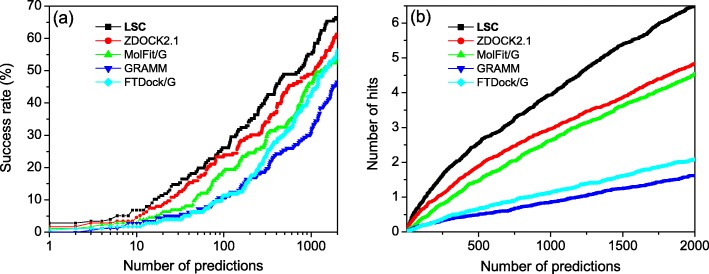
The success rate (**a**) and the average number of hits per case (**b**) as a function of the number of top predictions for our LSC-implemented docking program, ZDOCK 2.1, MolFit/G, GRAMM, and FTDock/G for unbound docking on the protein-protein docking benchmark 4.0. The results of MolFit/G, GRAMM, and FTDock/G were taken from our previous study [22]

The success rate and the average number of hits per case for unbound docking of our LSC method are shown in Fig. 3. Tables 1 and 2 list values of success rates and average number of hits for several certain numbers of top predictions. For comparison, Fig. 3 and Tables 1-2 also give the corresponding results of the other four docking programs, ZDOCK 2.1 [47], MolFit/G [45], GRAMM [42], and FTDock/G [41]. From Fig. 3a we can see that our LSC again performed the best among the five scoring methods in binding mode predictions. For top 1, 10, 100, and 1000 predictions, LSC obtained a success rate of 2.84, 6.82, 26.14, and 55.11%, respectively, followed by 1.71, 4.55, 23.86, and 48.86% for ZDOCK 2.1, 1.14, 2.84, 18.75, and 46.02% for MolFit/G, 0.00, 2.84, 10.80, and 30.68% for GRAMM, and 0.57, 1.71, 11.36, and 42.61% for FTDock/G (Table 1). As for the average number of hits, our LSC also achieved the best performance among the five methods and obtained an average of 0.83, 2.54, 3.94, and 6.51 hits when the top 100, 500,1000, and 2000 predictions were considered, followed by 0.61, 1.89, 2.96, and 4.84 hits for ZDOCK 2.1, 0.36, 1.47, 2.62, and 4.55 hits for MolFit/G, 0.18, 0.51, 0.86, and 1.63 hits for GRAMM, and 0.18, 0.65, 1.13, and 2.08 hits for FTDock/G (Table 2).

Comparing the results of bound and unbound docking also shows that although the absolute performances of unbound docking are worse than those of bound docking for all five scoring methods, the relative improvement of LSC over the other scoring functions is more significant for unbound docking compared to that for bound docking (Figs. 2 and 3). For example, the success rate increases 21.4% from 42.61% of the second-best ZDOCK 2.1 to 51.71% of the best LSC for bound docking, while the success rate increases 49.9% from 4.55% of the second-best ZDOCK 2.1 function to 6.82% of the best LSC for unbound docking for top 10 predictions. The significantly better performance of our LSC scoring method for unbound docking again demonstrates the robustness of our scoring method for describing shape complementarity in docking unbound structures.

### Performance by complex types

**Fig. 4:**
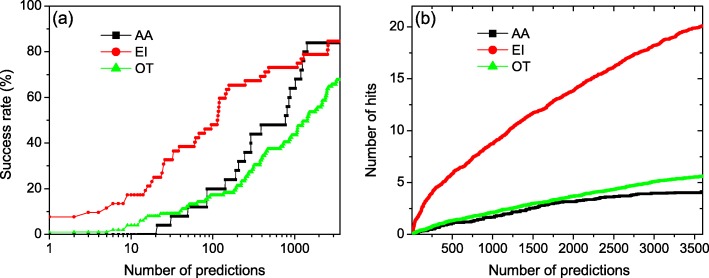
The success rate (**a**) and the average number of hits per target (**b**) as a function of the number of top predictions for our LSC-implemented docking program on three categories of complexes including Enzyme/Inhibitor(EI), Antibody/Antigen (AA), and Other types (OT)

It has been shown that different types of complexes may exhibit intrinsically different interaction characteristics [20]. To investigate the effect of different complex types on our docking results, Fig. 4 shows the success rates and the average number of hits per target as a function of the number of top predictions for three types of targets: 52 enzyme/inhibitor cases (EI), 25 antibody/antigen cases (AA), and 99 other types (OT) of cases. From the figure we can see that overall the enzyme/inhibitor type have many more hits than the antibody/antigen and other types of complexes. For example, our docking program yielded an average of 13.8 hits per complex for enzyme/inhibitor when the top 2000 predictions were considered, compared to 3.2 hits for antibody/antigen and 3.7 hits for other types, respectively. For the success rate, the EI cases always performed better than OT cases and also better than AA cases until the top 1268 predictions were considered. After the top 1268 predictions, the success rate of AA type increases fast and then surpasses that of the EI type. Finally the success rates of AA and EI types become comparable. This phenomenon can be explained by previous findings that enzymes and their inhibitors have coevolved resulting in a highly complementary interface [78]. However, the antibody/antigen and other types of complexes do not necessarily form the best possible binding interface. For example, many different antibodies are produced by the immune system in response to an antigen, while some quite poorly bind with the antigen. Therefore, the EI complexes tend to be easier targets due to their better shape complementarity between two partners and have more hits, compared to the AA and other types of complexes. Therefore, the near-native ones of EI complexes will get a higher shape complementarity and rank higher than AA or OT cases. However, this result does not indicate that the shape complementarity is not important for the complexes of AA type. As we can see from Fig. 4, there are still many near-native hits for AA complexes in the low-ranking predictions, and the final success rate of AA type is almost the same as that of the EI type. This indicates that our docking program can still sample the near-native hits for many AA complexes, although most of them have a low ranking. Therefore, using shape complementarity as the first filter is still an efficient docking strategy even for AA complexes and in this case, we should consider more predictions for post-docking approaches, compared to the EI cases.

### Computational efficiency

**Fig. 5:**
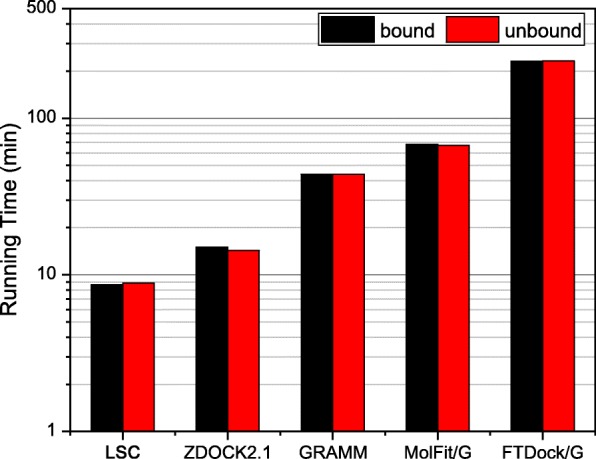
The average running time for both bound and unbound docking of our LSC docking program and four other shape-based docking algorithms on the 176 targets of the protein docking benchmark 4.0, where the running times of ZDOCK2.1, MolFit/G, GRAMM, and FTDock/G were obtained based on our previous study [22]

Besides the docking performance, computational efficiency is also an important index to evaluate the docking algorithm, especially when the computational resources are insufficient. The average running times of LSC and other four shape-based docking algorithms for both bound and unbound docking tested on the protein-protein docking benchmark 4.0 are shown in Fig. 5, where the running times of ZDOCK 2.1, GRAMM, MolFit/G, and FTDock/G were extracted from our pervious study [22] and adjusted for the present new hardware by using ZDOCK2.1 as the reference. It can be seen from Fig. 5 that our algorithm LSC consumes the least time with an average of 8.7 min for a bound docking run and 8.9 min for a unbound docking run, compared to 15.0 and 14.3 min for ZDOCK 2.1, 43.8 and 43.9 min for GRAMM, 68.3 and 67.1 min for MolFit/G, and 231.0 and 231.8 min for FTDock/G. The computational efficiency for an FFT-based docking algorithm is mainly determined by the sizes the proteins to be docked. As the size of unbound structures is very similar to that of the bound structures for each target, the average running times for bound and unbound docking are almost the same for each docking method. The least running time of our LSC in both bound docking and unbound docking demonstrated its highest computational efficiency among the five shape-based docking algorithms.

## Discussion

To consider the long range interactions of protein atoms in shape complementarity, we have developed an FFT-based docking program with a long-range pairwise shape-based scoring function (LSC) through an exponential form. In many cases, only the shape complementarity scoring function is necessary for a docking calculation. The reason for this is that as the binding mode input for other post-docking refinement/rescoring approaches, a protein docking algorithm should offer a set of widely sampled protein-protein binding poses that do not bias towards to any special interaction energies. The shape complementarity is exactly the choice for such requirements. In addition, the shape is such a simple descriptor for a protein and therefore the shape complementarity will put less limitation on filtering the generated binding modes so that the docking program can yield diversely sampled protein binding orientations.

Shape representation is also the foundation of other energy terms for a scoring function. For example, in FFT-based docking algorithms, all the energy terms like electrostatic interactions and hydrogen bonding should be mapped onto discrete grids that characterize the proteins. During the energy mapping, the scoring function may lose part of the accuracy, where shape representation of the protein has a critical impact on the accuracy of energy mapping. Therefore, the present LSC lays a good basis for the characterization of other energy terms on grids.

Exact comparison between our protein docking program and other algorithms is not feasible due to different sampling and/or scoring methods. One closest docking program to our docking model is the ZDOCK 2.1 which uses a pairwise shape complementarity scoring function (PSC) for protein-protein docking [47]. Therefore, we used ZDOCK 2.1 as a reference to verify our FFT-based algorithm with LSC, although we also listed the results of other three shape-based docking methods, MolFit/G, GRAMM, and FTDock/G. As shown in the docking results, our LSC-based docking program performed better than ZDOCK 2.1 in both the success rate and the average number of hits per target for bound and unbound docking. As described in the methods, our LSC considered the effects of more distant grids than PSC and GSC to partly consider the long range effect of the interactions such as van der Waals interactions. The better performance of our docking program compared to ZDOCK 2.1 indicated the importance of considering long-range interactions in shape complementarity.

The different performances on three types of complexes are consistent with the previous findings that the interface of antigen-antibody complex is usually small and has a poor shape complementarity. Therefore, their near-native hits are often within the low-ranking predictions. Accordingly, for post-docking purposes, if the computing resource is limited, it is suggested that the top 100 predictions are kept for EI complexes while the top 1000 predictions are retained for AA and OT complexes, which correspond to a success rate of about 50% in the present LSC-based docking. However, if possible, considering the all 4392 predictions is recommended.

## Conclusions

We have developed a new pairwise shape complementarity scoring function to take into account the effects of long-range interactions in protein-protein docking. The protein grid is divided into a protein core, a near-surface layer, a surface layer and outside space for the long-range shape-based scoring (LSC) function. The repulsion component for the near-surface layer is the sum of the contributions of neighboring core atoms in the protein, and the favorable component for the surface layer comes from the near-surface and core atoms. Our FFT-based docking program with LSC was extensively tested on the protein docking benchmark 4.0 by the Weng group for both bound docking and unbound docking. Compared to other four shape-based docking programs, ZDOCK 2.1, MolFit, GRAMM, and FTDock, our LSC significantly improved the docking performance in both the success rate and the average number of hits. The significantly better performance of LSC compared to other shape-based scoring functions for bound and unbound docking suggests the accuracy and robustness of our method in characterizing shape complementarity. The different performances on three types of complexes (AA, EI, and OT) are consistent with the previous findings. It is suggested that if the computing resource is limited, the top 100 predictions are kept for EI complexes while the top 1000 predictions are retained for AA and OT complexes for post-docking processes, though using all the 4392 binding modes are recommended if possible.

## Data Availability

The LSC-implemented docking approach is freely available as part of the HDOCK web server at http://hdock.phys.hust.edu.cn/.

## Abbreviations

*L*_rmsd_: ligand root mean square deviation
AA: antibody/antigen
EI: enzyme/inhibitor
FFT: fast-Fourier transform
GSC: grid-based shape complementarity scoring function
LSC: long-range shape complementarity scoring function
OT: other types
PDB: Protein Data Bank
PSC: pairwise shape complementarity scoring function
RMSD: root mean square deviation
